# Coping Style Modifies General and Affective Autonomic Reactions of Domestic Pigs in Different Behavioral Contexts

**DOI:** 10.3389/fnbeh.2017.00103

**Published:** 2017-05-30

**Authors:** Annika Krause, Birger Puppe, Jan Langbein

**Affiliations:** ^1^Institute of Behavioural Physiology, Leibniz Institute for Farm Animal Biology (FBN)Dummerstorf, Germany; ^2^Behavioural Sciences, Faculty of Agricultural and Environmental Sciences, University of RostockRostock, Germany

**Keywords:** coping style, autonomic nervous system, affect, telemetry, domestic pig

## Abstract

Based on individual adaptive strategies (coping), animals may react differently to environmental challenges in terms of behavior and physiology according to their emotional perception. Emotional valence as well as arousal may be derived by measuring vagal and sympathetic tone of the autonomic nervous system (ANS). We investigated the situation-dependent autonomic response of 16 domestic pigs with either a reactive or a proactive coping style, previously selected according to the backtest which is accepted in piglets to assess escape behavior. At 11 weeks of age, the pigs were equipped with an implantable telemetric device, and heart rate (HR), blood pressure (BP) and their respective variabilities (HRV, BPV) were recorded for 1 h daily over a time period of 10 days and analyzed in four behavioral contexts (resting, feeding, idling, handling). Additionally, the first minute of feeding and handling was used for a short-term analysis of these parameters in 10-s intervals. Data from day 1–3 (period 1) and day 8–10 (period 2) were grouped into two separate periods. Our results revealed general differences between the coping styles during feeding, resting and handling, with proactive pigs showing higher HR compared to reactive pigs. This elevated HR was based on either lower vagal (resting) or elevated sympathetic activation (feeding, handling). The short-term analysis of the autonomic activation during feeding revealed a physiological anticipation reaction in proactive pigs in period 1, whereas reactive pigs showed this reaction only in period 2. Food intake was characterized by sympathetic arousal with concurrent vagal withdrawal, which was more pronounced in proactive pigs. In contrast, neither coping style resulted in an anticipation reaction to handling. Vagal activation increased in reactive pigs during handling, while proactive pigs showed an increase in sympathetically driven arousal in period 2. Our findings confirm significant context-related differences in the general autonomic reaction of pigs with different coping styles. Additionally, the two coping styles differ in their affective appraisal over the time course of the experiment, underlining the importance of taking individual differences into account when studying affect and emotion in humans and animals.

## Introduction

Individual reaction patterns have been intensively investigated in different species in behavioral and evolutionary ecology in recent years. Consistent individual differences in the average level of behavior across time and contexts are commonly referred to as “animal personality” and exist in a range of animal taxa (Dall et al., 2004; Réale et al., 2007; Biro and Stamps, 2008; Dingemanse et al., 2010). It has been widely recognized that such individual variations play an important role in health and disease in humans and in animals (Koolhaas et al., 1999). One key element in this context is “coping”, which comprises a set of behavioral and physiological characteristics of an individual trying to master the challenge of an aversive situation (Lazarus, 1966). From the viewpoint of stress research, generally two fundamental coping patterns may be distinguished in animals: a “proactive or active pattern” on the one hand and a “reactive or passive pattern” on the other hand (Henry and Stephens, 1977). Despite domestication, targeted selection, genetic modification and inbreeding, the same coping strategies can also be observed in laboratory and farm animals. Studies in rodents basically distinguish between proactive and reactive coping (Koolhaas et al., 1986 [rats]; van Oortmerssen et al., 1985 [mice]) and studies in fish and birds often use the terms shyness and boldness (Wilson et al., 1994). In pigs, the concept of coping is also supported, although the extremes in the population do not represent distinct categories of pigs (Ruis et al., 2002; Zebunke et al., 2015). Studies focusing on coping styles in animals suggest that the proactive response is characterized by a typical active fight-flight response in aversive situations which is associated with territorial control, aggression and risk-taking (Cannon, 1929; Benus et al., 1990; Mount and Seabrook, 1993; Koolhaas et al., 1999). Conversely, the reactive coping style shows a conservation withdrawal response in aversive situations, which is characterized behaviorally by immobility and low levels of aggression (Engel and Schmale, 1972). Generally, the two coping styles differ fundamentally in their general adaptive response patterns in reaction to challenges (Koolhaas et al., 1999).

These challenges, just as other types of internal and external stimuli, also exert physiological effects, as they affect the activity of the autonomic nervous system (ANS). The perception of a challenging situation or stimulus leads to specific hierarchical, neurophysiological changes that originate from the brain, which is the key organ in reacting to and coping with stress. The first affective reaction was described as a fast neurophysiological reaction to prepare the body for action in a given circumstance (Posner et al., 2005). This process occurs subcortically and is therefore unconscious. Further processing of affect recruits additional brain systems and results in the experience of a subjective feeling or emotion, based on neocortical processing (Russell, 2003). This distributed neural circuitry determines what is threatening and stressful to the individual. Brain regions such as the amygdala, the hypothalamus and the brain stem are essential for autonomic responses to stressors, as they control the activity of sympathetic and vagal tone at the heart and the vessels, which affects sinoatrial node activity and blood pressure (BP; Carretié et al., 2009). The complex interaction between the two parts of the ANS produces complex variations in heart rate (HR) and BP. HR variability (HRV) as a result of this rhythmic oscillation is widely used as an indicator of autonomic function in the analysis of physiological signals in humans and animals (Task Force of the European Society of Cardiology and the North American Society of Pacing and Electrophysiology, 1996; von Borell et al., 2007). HRV parameters provide information about the complex interaction between the two branches of the ANS or about vagal regulatory activity. In contrast, BP fluctuations deliver information about sympathetic control, as Hedman et al. (1992) found that blocking sympathetic nerve transmission decreased all components of BP variability (BPV), whereas vagotomy decreased HRV but did not influence BPV. Recent autonomic blockade studies in pigs support this assumption (Poletto et al., 2011). Emotional perceptions of different natures can induce different shifts of autonomic balance, towards either a sympathetic or vagal prevalence. Therefore, analyzing cardiovascular dynamics is regarded as a suitable approach to draw conclusions about changes in sympathovagal balance related to ongoing appraisal processes and emotional states (Porges, 2003; Boissy et al., 2007; von Borell et al., 2007; Reefmann et al., 2009). From human research, it is known that emotional affective states can be defined in terms of two fundamental underlying dimensions. Emotional experiences are valenced, ranging from positive to negative and they also vary in the degree of arousal ranging from low to high (Russell and Barrett, 1999; Burgdorf and Panksepp, 2006; Mendl et al., 2010). Regarding autonomic regulation, researchers have investigated the relationship between the two dimensions and the activity of the two branches of the ANS. Several studies demonstrate a link between cardiac vagal tone and psychological components in negative contexts such as anxiety (Sleigh and Henderson, 1995) or panic attacks in humans (Friedman and Thayer, 1998) as well as in positive contexts, for example, in humans watching an emotionally positive film (Matsunaga et al., 2009) or in pigs showing a state of positive arousal when they were individually called to feeding in an operant conditioning paradigm (Zebunke et al., 2011). These studies emphasize that positive emotions significantly increase vagal tone, whereas the opposite occurs with negative emotions. Therefore, vagal tone is assumed to reflect the valence dimension of affect. The arousal dimension was investigated in numerous studies in different species describing sympathetic activation (de Boer et al., 1990) and a rise in HR (Farah et al., 2004) in reaction to negative situations, whereas other authors describe similar results in positive contexts such as appetitive conditioning in primates (Braesicke et al., 2005). The fact that both positive and negative affective states may increase sympathetic tone supports the assumption that it may represent the arousal dimension of affect (Yeates and Main, 2008; Mendl et al., 2010).

In recent years, advances in technology allow the use of mobile, fully implantable telemetric techniques that are able to verify a number of cardiovascular parameters. Usually, such systems find application in the context of pharmacological methods and cardiovascular diseases (Gelzer and Ball, 1997; Stubhan et al., 2008). Using the domestic pig as a suitable animal model, previous studies have shown that an implantable telemetric system, capable of measuring electrocardiogram (ECG) and intra-arterial BP simultaneously in free-ranging animals, resulted in the reliable assessment of both branches of the ANS while physiological variables were precisely assessed with minimal disturbance to the animal (Krause et al., 2015, 2016). Differences between the coping styles were found in numerous studies in the context of behavior (Koolhaas et al., 1999), physiology (Hessing et al., 1994a; Ruis et al., 2000), immune responses (Oster et al., 2015) and genetic variants (Ponsuksili et al., 2015). Coping behavior associated with detailed autonomic regulation remains largely understudied (for review, see Koolhaas et al., 1999, 2010; de Boer et al., 2017). In the present article, we used behavioral differences, resulting in proactive or reactive coping patterns, to identify phenotypes related to autonomic responses during different behavioral contexts. Autonomic reaction was investigated using an implantable telemetric device in free-ranging animals to assess both branches of the ANS. The first aim of the present study was the assessment of the general autonomic reaction of pigs with different coping styles in different behavioral contexts to identify the link between coping patterns and their respective autonomic reaction in relation to behavior. The second aim was the determination of short-term affective autonomic reactions of pigs during feeding and handling to reveal differences in their affective appraisal by characterizing the valence and arousal dimensions of affect in relation to coping styles. We expected context-related differences between the coping styles in their general autonomic reaction and in their affective appraisal depending on the respective behavioral situation.

## Materials and Methods

### Ethical Statement

Animal care and all experimental procedures were in accordance with the German welfare requirements for farm animals and the ASAB/ABS Guidelines for the Use of Animals in Research (Anonymous, 2016). All procedures involving animal handling and treatment were approved by the Committee for Animal Use and Care of the Ministry of Agriculture of Mecklenburg-Vorpommern, Germany (Ref. Nr. 7221.3-1.1-037/12).

### Animals and Housing

In three identical replicates, a total of 16 female domestic pigs (*Sus scrofa*, German Landrace) from the experimental facilities for swine of the Leibniz Institute for Farm Animal Biology (FBN) in Dummerstorf were studied from 11 weeks of age. For each replicate, eight piglets were selected on the basis of their previously tested behavioral response in the backtest in order to describe their coping style as either proactive or reactive. Selected piglets were weaned at 28 days of age and kept in groups of eight (4 proactive, 4 reactive piglets) until 10th week of life, when pigs were moved to the experimental housing facilities of the institute. They were housed in individual pens (2.67–3.31 m^2^) with a solid and partially slatted floor and visual, olfactory, acoustic and partial tactile contact with conspecifics (snout contact) in the adjacent pens. The pens were separated by means of transparent walls (acrylic glass) that enabled full sight of the adjacent pens. A metal food trough with a lockable cover (23 × 42 cm) was fitted next to the entrance of the pen. The animals were fed twice a day and had free access to water (nipple drinker). The individual pens were located in an experimental room, which provided an area for technical equipment, an electronic balance and pig food including the buckets. The temperature was held constant at 20°C. During 1 week of acclimatization, the pigs were weighed and handled three times daily for 1 h to socialize and habituate them to human contact and equipment monitoring. At 11 weeks of age (approximately 30 kg weight), 4–6 pigs in each replicate underwent surgery for the implantation of a telemetric device capable of recording ECG, BP and body temperature (Krause et al., 2016). After surgery, pigs were returned to their familiar individual pen for the following 3 weeks. Based on technical issues concerning the telemetric system, two transmitters did not transfer any cardiovascular data. After these animals were excluded from further analysis, the data of 14 pigs were included in the study.

### Backtest and Classification

The backtest was carried out at the ages of 5, 12, 19 and 26 days, according to the methodology described by Zebunke et al. (2015). The piglets were housed in a commercial farrowing pen measuring 2 × 3 m with a crate and creep area. The pens included a fully slatted floor (except for the creep area, which contained a heating panel and a metal plate for the sow’s front legs). A V-shaped cradle fixed on a table was positioned in front of each farrowing pen and used for the backtest. Each piglet was put in the cradle in a supine position on its back for 1 min. Three parameters were recorded: (i) latency (time span until the pig showed the first struggling attempt); (ii) total duration; and (iii) frequency of all struggling attempts within 60 s. The classification of piglets in either proactive or reactive coping styles was based on previously defined upper and lower quartiles of the recorded parameters to determine the respective classification ranges (latency: 5 and 35 s, duration: 5 and 25 s, frequency: 1 and 4; Oster et al., 2015; Ponsuksili et al., 2015). A total of 12 measures per piglet (three recorded parameters in four repetitions) were considered for classification into proactive (“high resisting”), reactive (“low resisting”) or doubtful. If the classification was statistically significant (binomial test), the piglet fell in one of the categories. For each replicate, 80 female piglets were tested in the backtest, while eight piglets (4 reactive and 4 proactive) were selected for the study and finally 4–6 pigs underwent surgery and were used for the experiment.

### Telemetric Equipment and Signal Monitoring

The telemetric system consisted of four components: the implantable device (transmitter), individual receivers, data acquisition device (Power Lab) and analysis software (LabChart) and was provided by Telemetry Research (Auckland, New Zealand) and ADInstruments (Oxford, UK). The implantable transmitter unit (Telemeter model TRM84PB, 90 mm × 45 mm × 10.5 mm, 64 g) measures and transmits ECG using two flexible biopotential leads (30 cm in length) and intra-arterial BP using a fluid-filled catheter (1 mm OD (3Fr) Catheter, Millar Instruments, Houston, TX, USA) via telemetry from within the animal. A temperature sensor embedded in the implant case was used to measure the animal’s body temperature. Each implanted transmitter relayed digital signals to a receiver unit using an individual frequency. A switch at the receiver enabled the transmitter to be turned on and off *in vivo* without affecting the animal. The receivers were connected by wire to two digital data acquisition systems (PowerLab) that allowed the simultaneous real-time recording of up to six pigs at a sampling rate of 2 kHz. The sampled data were stored and displayed on a PC and analyzed using LabChart Pro (Version 7.0, ADInstruments).

### Transmitter Implantation

For detailed information regarding surgery, please see Krause et al. (2016). Here, we give a brief overview of the implantation process. The pigs were fasted 12 h prior to surgery but allowed access to water. The animals were subsequently implanted with the telemetric device by the veterinarian of the institute under sterile conditions. Prior to surgery, the pigs were anesthetized in their home pen with a combination of xylazine 2 mg/kg (Xylariem, Riemser Arzneimittel) and ketamine 20 mg/kg (Ursotamin, Serumwerk) intramuscularly. For local anesthesia, procaine 4 mg/kg (Isocain, Selectavet, Dr. Otto-Fischer) was injected subcutaneously according to the planned incision lines. During surgery, general anesthesia was maintained using ketamine 4 mg/kg/h (Ursotamin, Serumwerk) and diazepam 0.4 mg/kg/h (Faustan, AWD-pharma) intravenously in a 5% glucose solution. The telemetric device was placed in a subcutaneous pouch at the left side of the neck. The positive electrode was positioned dorsal ~2 cm lateral to the left scapula, while the negative electrode was located lateral to the sternum. Both electrode tips were enclosed by muscle tissue. The pressure sensor was inserted into the left carotid artery. After completing surgery, pigs were returned to their respective home pens and allowed to recover under a heat lamp to maintain body temperature after surgery. They were observed continuously and body temperature, HR and BP were controlled every hour until they recovered consciousness and exhibited complete recovery from anesthesia. The pigs were administered a postsurgical dose of 50 mg/kg metamizole (Metapyrin, Serumwerk) and a combination of 20 mg/kg sulfadimidine and 4 mg/kg trimethoprim (Trimethosel, Selectavet, Otto-Fischer GmbH) every 24 h for 5 days. Skin sutures were removed on days 10 and 11 postoperatively.

### Data Acquisition

Behavior, ECG, BP and body temperature were recorded for 1 h daily from 08.00 a.m. to 09.00 a.m. over a time period of 10 days after surgical implantation of the transmitter. A familiar person entered the experimental room at 08.15 a.m. daily to feed and at 08.45 a.m. to handle the pigs in each individual pen (2 min each). The handling procedure started with entering the single pen. Once the animal had approached the familiar person, including tactile snout contact, it was gently stroked and scratched. Pig behavior was assessed using video cameras (Panasonic WV-CP500, EverFocus Endeavor SD + HD DVR) attached to each single housing pen and analyzed using the Observer XT (Version 11, Noldus, Wageningen, Netherlands). Cardiovascular data were calculated using LabChart. Mean HR and the short-term variation of HR (RMSSD = root mean of the squared distances of subsequent inter-beat-intervals, an indicator of vagal activation) were obtained from ECG recordings. Using the BP signal, systolic BP (SBP) and its standard deviation (SD_SBP_, indicative of sympathetic activation) were assessed.

#### Analysis of the General Autonomic Reaction in Different Behavioral Contexts

Cardiovascular data during four behavior categories, each 5 min in duration (recorded daily over a time period of 10 days), were chosen for analysis: resting (lying inactive during the prefeeding period), feeding (08.15 a.m.), idling (locomotion, exploration of floor or wall after the feeding period) and handling (08.45 a.m., 2 min).

#### Analysis of the Affective Autonomic Reaction during Feeding and Handling

Additionally, to evaluate affective appraisal in the pigs, a time span of 50 s each from the feeding and handling situations was chosen for more detailed analysis of the autonomic activity in 10-s intervals. For the feeding situation, we chose the time period from 20 s before feeding to 30 s after feeding. Time interval (TI) 1 included 10 s before a familiar person entered the room (TI 1: −20 to −10 s). Only pigs with active (idling) behavior during this TI were included in the analysis (pigs that were lying down were excluded) due to the large effect of physical activity on cardiovascular parameters. TI 2 (–10 to 0 s) included the 10 s immediately before feeding. The person entered the room, and prepared the buckets for feeding. During this TI, the pigs had the opportunity to develop an anticipation reaction over the time course of the experiment. With the beginning of TI 3, the pigs were fed. TI 3–5 involved food intake itself.

In the handling situation, the time span from 20 s before entering the single pen to 30 s after entering the single pen was chosen. TI 1 included the time before the familiar person was present (−20 to −10 s). Here, again, only pigs with active behavior were included in the analysis. During TI 2 (−10 to 0 s), the familiar person was standing in front of the single housing pen. With the beginning of TI 3, the person entered the single pen for handling. During TI 3, 4 and 5 (0–30 s), the person was present in the single pen. To assess the development of the autonomic reaction over the time course of the experiment in both repeated situations (feeding and handling), data from day 1–3 and from day 8–10 were grouped into two separate periods (period 1: day 1–3, period 2: day 8–10).

#### Approach Latencies in the Handling Context

Approach latencies which comprised the time from entering the single pen until first tactile snout contact towards the familiar person were recorded.

### Statistical Analysis

All statistical analyses were conducted using SAS (version 9.3, 2009, SAS Institute Inc., Cary, NC, USA). The normality of the distribution of each parameter was assessed using Kolmogorov-Smirnov tests. Where normality assumptions were not met, data were logarithmically transformed. The data were evaluated by analysis of variance (ANOVA) using the GLIMMIX procedure in SAS/STAT software. All analyses included the animal as a repeated factor and mean differences with a *p* < 0.05 were considered significantly different.

#### Analysis of the General Autonomic Reaction in Different Behavioral Contexts

The model for the measured parameters HR, RMSSD, SBP, SD_SBP_ and body temperature contained the main effects replicate (1–3), experimental day (1–10), behavior (resting, feeding, idling and handling), coping style (reactive, proactive) and their respective two-way interactions. Least squared means (LSMs) and their standard errors (SEs) were computed for each fixed effect in the model. All pairwise differences of these LSM were tested using the Tukey-Kramer method, the procedure for pairwise multiple comparisons with the best power. In addition, the slicediff option of the LSM statement of the GLIMMIX procedure was used for partitioned analyses of the LSM for the two-way interaction of coping style × behavior (i.e., comparisons between the coping styles within the respective behavioral context and vice versa).

#### Analysis of the Affective Autonomic Reaction during Feeding and Handling

The statistical model consisted of fixed effects of the replicate (1–3), period of the experiment (period 1, period 2), TI (1–5), coping style (reactive, proactive) and their respective interactions, including three-way interaction. LSM and SE were computed for each fixed effect in the model and all pairwise differences of these LSM were tested using the Tukey-Kramer method. For comparisons of the coping styles within each TI and each period (i.e., performing partitioned analyses of the LS-means for the interaction of period × TI × coping style), the slicediff option of the LSM statement of the GLIMMIX procedure was applied.

#### Approach Latencies in the Handling Context

Data on approach latency were analyzed for the repeated handling situation, using a model with the fixed factors replicate (1–3), period of the experiment (period 1, period 2), coping style (reactive, proactive) and the interaction of coping style × period. LSM and SE were computed for each fixed effect in the model. Pairwise differences between LSMs were evaluated using the Tukey-Kramer method.

## Results

### Impact of Surgical Procedure on Cardiovascular Parameters and Body Temperature in a 10-Day Convalescence Period

Within the different behavioral contexts, the cardiovascular parameters did not change over the 10-day period after the surgical procedure (interaction of the fixed effects day × behavior: HR: *F*_(27,397)_ = 0.94, *p* = 0.5; RMSSD: *F*_(27,338)_ = 0.23, *p* = 0.99; SBP: *F*_(27,321)_ = 0.74, *p* = 0.83, SD_SBP_: *F*_(27,301)_ = 1.1, *p* = 0.34). Body temperature was found to be affected by the day (*F*_(9,108)_ = 4.4, *p* < 0.001), whereas the coping style did not have any effects (*F*_(1,10)_ = 0.66, *p* = 0.43). The lowest body temperature was measured at day 1 (39.3 ± 0.06°C), but an increase was found at day 3 (39.6 ± 0.06°C), followed by a stable course until the end of the experiment. After the postsurgical medication was concluded at day 5, body temperature remained constant. All pigs showed an uncomplicated healing process, as they appeared in good health with fast wound healing, normal grooming behavior and the absence of any inflammation reactions or complications.

### General Autonomic Reaction in Different Behavioral Contexts

**Figure 1:**
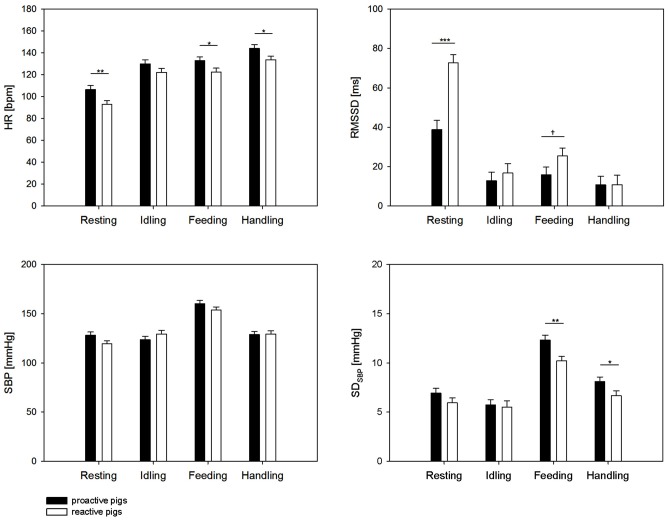
Heart rate (HR [bpm]), root mean square of successive differences (RMSSD [ms]) systolic blood pressure (SBP [mmHg]) and standard deviation of SBP (SD_SBP_ [mmHg]) during 5 min of resting, idling, feeding and 2 min of handling. Black bars: proactive pigs, white bars: reactive pigs. Data are presented as least squared means and standard errors (LSM ± SE). Significant differences between the coping styles during the respective behavior are indicated by asterisks (*p < 0.05, **p < 0.01, ***p < 0.001) and trends are indicated by † (^†^*p* < 0.1).

We found a significant effect of the interaction of coping style × behavior on HR (*F*_(3,209)_ = 3.7, *p* < 0.05), RMSSD (*F*_(3,343)_ = 9.09, *p* < 0.001), SBP (*F*_(3,322)_ = 6.9, *p* < 0.001) and a tendency on SD_SBP_ (*F*_(3,303)_ = 2.3, *p* < 0.1). SD_SBP_ was significantly affected by the factor behavior (*F*_(3,303)_ = 108.9, *p* < 0.001). As shown in Figure 1, the results revealed a significantly higher HR in proactive pigs during resting (*p* < 0.01), feeding (*p* < 0.05) and handling (*p* < 0.05) compared to reactive pigs. The difference in HR during resting was accompanied by lower RMSSD values in proactive than reactive pigs (*p* < 0.001). During feeding, the differences in HR between the coping styles were associated with higher SD_SBP_ (*p* < 0.01) and a trend to lower RMSSD (*p* < 0.1) in proactive pigs. The elevated HR in proactive pigs (*p* < 0.05) during handling was exclusively accompanied by elevated SD_SBP_ values (*p* < 0.01). During idling, no differences in autonomic regulation were found between the coping styles (all parameters: *p* > 0.1).

### Affective Autonomic Reaction during Feeding

**Figure 2:**
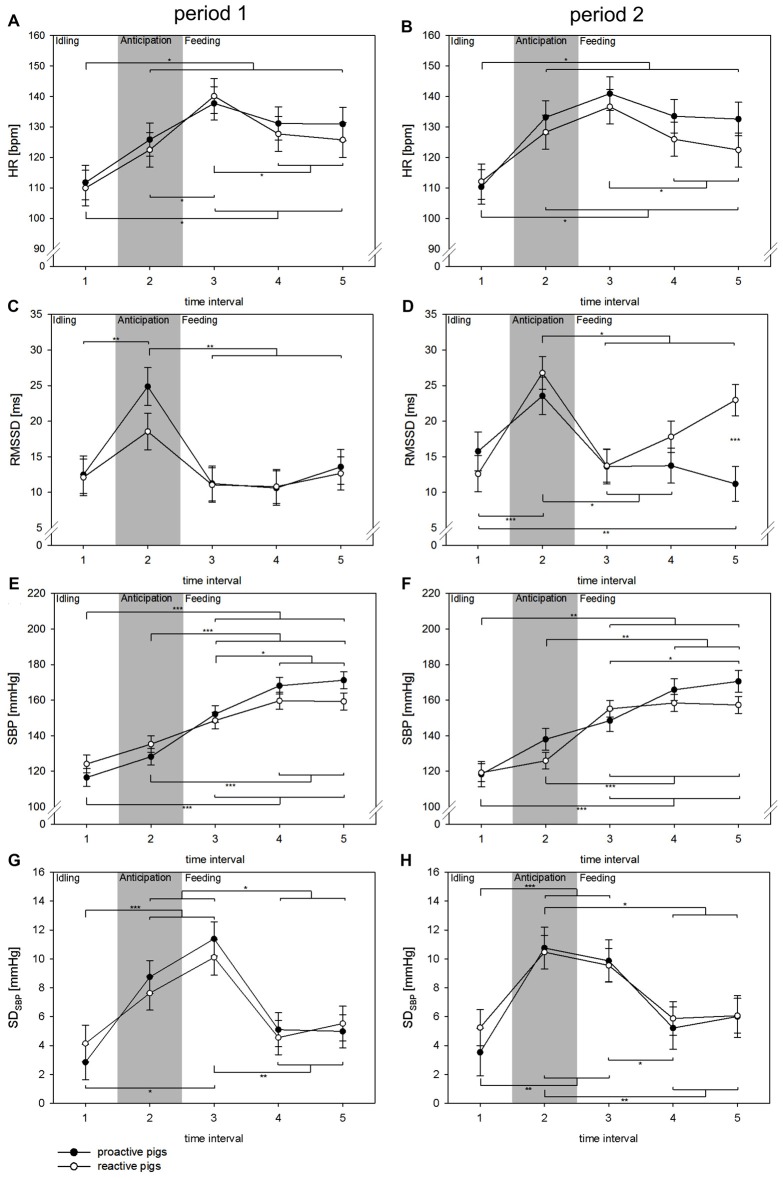
Changes in HR (**A,B** [bpm]), RMSSD (**C,D** [ms]), SBP (**E,F** [mmHg]) and SD_SBP_ (**G,H** [mmHg]) in time intervals (TIs) of 10 s (TIs 1–5) in experimental period 1 (left: **A,C,E,G**) and experimental period 2 (right: **B,D,F,H**) in the context of feeding. Black dots: proactive pigs, white dots: reactive pigs. Data are presented as LSM ± SE. Significant differences (*p < 0.05, **p < 0.01, ***p < 0.001) between TIs are shown above (proactive pigs) and below (reactive pigs) the dots. Differences between the coping styles in each TI are presented between the dots. Gray area: the person is entering the room and preparing food buckets.

The detailed analysis of the feeding situation showed that all cardiovascular parameters changed significantly over the TIs (HR: *F*_(4,245)_ = 42.9, *p* < 0.001; RMSSD: *F*_(4,193)_ = 15.6, *p* < 0.001; SBP: *F*_(4,196)_ = 74.8, *p* < 0.001, SD_SBP_: *F*_(4,129)_ = 14.7, *p* < 0.001; Figure 2). In period 1, proactive pigs showed an increase in HR in anticipation of food (TI 2) when the person entered the room to prepare the buckets. This effect was consistent over the periods and was accompanied by a parallel increase in RMSSD (*p* < 0.01) and SD_SBP_ (*p* < 0.001) in proactive pigs. Similar autonomic activation was found in reactive pigs, but not until period 2. In period 2, reactive pigs developed higher RMSSD (*p* < 0.001) and SD_SBP_ values (*p* < 0.01) during TI 2 compared to period 1. With the beginning of feeding in TI 3 (0–10 s), HR rises to its maximum in both coping styles and both periods. The period was found to have an effect on RMSSD (*F*_(1,208)_ = 7.7, *p* < 0.01). RMSSD showed a general increase in period 2 compared to period 1 (*p* < 0.001). This effect was primarily accompanied by higher RMSSD values in reactive pigs compared to proactive pigs (*p* < 0.001) during food intake (TI 5) in period 2. Nevertheless, this effect was not reflected in HR.

### Affective Autonomic and Behavioral Reactions during Handling

Significant effects of the interaction of period × coping style (*F*_(1,68)_ = 6.1, *p* < 0.05) were found on the approach latency towards the familiar person in the pen. Reactive pigs showed longer latencies to approach the person in period 1 compared to proactive pigs (*p* < 0.05) and compared to period 2 (*p* < 0.001), as shown in Table 1. The difference between the coping styles disappeared in period 2.

**Table 1:** Approach latencies: time (s) from a familiar person entering the individual pen until the pig has approached and touched the person with the snout in period 1 (first 3 days of experiment) and period 2 (last 3 days of experiment).

	Period 1	Period 2
Proactive pigs	3.4 ± 1.5^a^	2.4 ± 1.5^a^
Reactive pigs	9.5 ± 1.5^b^	2.2 ± 1.5^a^

*Data are presented as least squared means and standard errors (LSM ± SE). Different letters (a, b) indicate statistically significant differences (*p* < 0.05) across lines (coping styles) and columns (periods)*.

**Figure 3:**
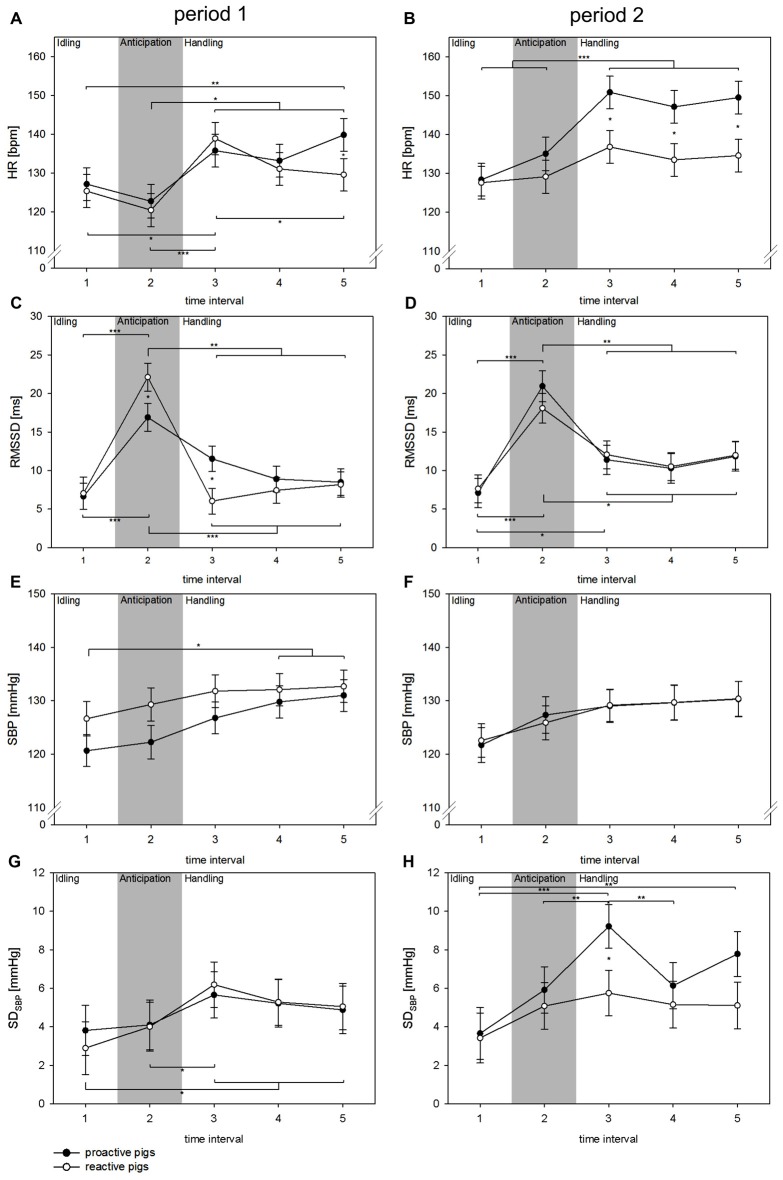
Changes in HR (**A,B** [bpm]), RMSSD (**C,D** [ms]), SBP (**E,F** [mmHg]) and SD_SBP_ (**G,H** [mmHg]) in TIs of 10 s (TIs 1–5) in experimental period 1 (left: **A,C,E,G**) and experimental period 2 (right: **B,D,F,H**) in a handling context with a familiar person. Black dots: proactive pigs, white dots: reactive pigs. Data are presented as LSM ± SE. Significant differences (*p < 0.05, **p < 0.01, ***p < 0.001) between intervals are shown above (proactive pigs) and below (reactive pigs) the dots. Differences between the coping styles in each TI are presented between the dots. Gray area: the person is standing in front of the single pen for 10 s.

Concerning the autonomic reaction during handling, the fixed factor TI showed a significant effect on all parameters (all: *p* < 0.001). The interaction of period × coping style was found to have a significant impact on HR (*F*_(1,379)_ = 7.7, *p* < 0.01) and SD_SBP_ (*F*_(1,142)_ = 4.9, *p* < 0.05), whereas the interaction of TI × period × coping style was significant in terms of RMSSD (*F*_(4,315)_ = 2.6, *p* < 0.05). Additionally, the interaction of TI × coping style showed a significant effect on HR (*F*_(4,376)_ = 3.3, *p* < 0.05). As shown in Figure 3, a strong increase in RMSSD (*p* < 0.05) was found at the moment the person was standing in front of the single pen (TI 2) in period 1. This was more pronounced in reactive pigs than in proactive pigs (*p* < 0.05). In both coping styles, no related changes in SD_SBP_ or HR were found in this TI. In period 2, RMSSD also increased during TI 2, but the differences between the coping styles disappeared in this TI. As the person entered the single pen for handling (TI 3), HR increased markedly in period 1 in both coping styles (proactive: *p* < 0.05, reactive: *p* < 0.001) accompanied by a strong withdrawal in the RMSSD, which was more pronounced in reactive pigs (TI 2 vs. TI 3: proactive: *p* < 0.01, reactive: *p* < 0.001) and a rise in SD_SBP_ only in reactive pigs (*p* < 0.05). In period 2, reactive pigs showed an elevated RMSSD during handling (TI 3) compared to period 1 and compared to TI 1, before the person was present. This effect was also reflected in HR, which remained constantly low after the person had entered the single pen in period 2. Proactive pigs showed a strong increase in SD_SBP_ and HR in period 2 during handling compared to period 1 (*p* < 0.01) and compared to reactive pigs (*p* < 0.05).

## Discussion

To our knowledge, this is the first study analyzing both branches of the ANS during different behavioral situations in regard to different coping styles in domestic pigs. Our study investigated the general autonomic activity as well as the short-term affective autonomic reaction of different coping styles to characterize the emotional valence and arousal dimensions of affect. Our study demonstrates that the determination of individual coping styles, which were classified on the basis of a proactive or reactive behavioral response in a repeated backtest, revealed different autonomic responses in selected behavioral contexts. Additionally, the coping styles also differed in their affective appraisal in two repeated situations over the time course of the experiment, as seen from different autonomic activation.

### General Autonomic Reaction in Different Behavioral Contexts

In the present study, the pigs’ coping style was related to differences in the ANS activity in three of four behavioral contexts. The results indicated lower HR accompanied by higher vagal activity during resting in reactive pigs than in proactive ones. High vagal tone has been linked to efficient autonomic activity, which enables the individual to increase its response to physiological and environmental challenges (Friedman and Thayer, 1998). In humans, resting autonomic activity was found to be associated with several behavior traits such as cooperative behavior (Beffara et al., 2016), chronic aggression (Mawson, 2009) and impulsiveness (Mathias and Stanford, 2003). In line with our results, higher resting HR and lower vagal activity was found in impulsive cows compared to reserved individuals (Kovács et al., 2015). Similar effects were also found in the passive coping style of hens (low feather pecking line), characterized by a strong cardiac parasympathetic response during manual restraint (Korte et al., 1999). In contrast, during 5 min of feeding and handling, the elevated HR in proactive pigs was accompanied by stronger sympathetic activation compared to reactive pigs. This finding supports the assumption that proactive coping is associated with higher activity of the sympatho-adreno-medullary system (Cannon, 1929). Several studies describe higher sympathetic reactivity in proactive coping in different species and different stressful situations (Korte et al., 1997 [chicken]; Fokkema et al., 1995 [rats]). Higher HR in proactive pigs was also demonstrated during the backtest in a study by Hessing et al. (1994a). However, the authors could only indirectly conclude that the tachycardia demonstrated arousal of the sympathetic system, as they did not record specific measures for either parasympathetic or sympathetic activation. A study from Désiré et al. (2004) found increased HR in lambs in reaction to the sudden presentation of an object. During this increase in HR, there was no modification of its variability, as seen in the stability of RMSSD, which reflects vagal activity. The authors concluded that there had been a compensatory reaction by an increase in the activity of the sympathetic branch, as there is no reliable HRV indicator of the activity of the sympathetic branch. By means of the novel telemetric technique used in our study, the simultaneous measurement of ECG and BP and the subsequent calculation of HRV and BPV enabled the quantification of both, parasympathetic and sympathetic activity in free-ranging pigs.

### Affective Autonomic Reaction during Feeding

As access to food is highly intrinsically motivating in pigs (Day et al., 1995), any stimulus in the context of food is considered biologically relevant for these animals. In the current study, autonomic arousal accompanies the anticipation as well as the consumption of food. Owing to restricted feeding in this study, it is likely that the pigs were highly motivated to feed. The pigs learned that the sight of food combined with the acoustic signal of preparing the food-pellets in buckets led to access to that food soon after. This state of high motivation paired with the resulting attention of the pigs towards the food buckets induced sympathetic activation, which indicated a high state of arousal. Concomitantly, we found a parallel increase in vagal activation. Similar autonomic changes were also found in monkeys (Braesicke et al., 2005) during the sight of highly preferred food. Proactive pigs developed this anticipatory reaction in period 1, whereas the reaction did not develop in reactive pigs until period 2. This finding supports results in maze experiments in rodents showing that animals with proactive and reactive coping strategies differ in the degree to which their behavior is guided by environmental cues (Koolhaas et al., 1999, 2010). The proactive coping style is characterized by actions that are principally based on predictions. This is in contrast to the reactive coping style, which is described to respond more flexibly to changing environmental stimuli. Studies in pigs confirmed this fundamental difference in cue dependency between the two coping styles and found that proactive coping resulted in the faster development of routines than reactive coping (Bolhuis et al., 2004).

During the consumption of food, the rise in HR and sympathetic activity was present from the beginning of the experiment (period 1) in both coping styles. In period 2, reactive pigs showed higher vagal activation, which resulted in a decrease in HR during the consumption of food. The activation of vagal tone as a physiological reaction to feeding has already been described in humans (Porges, 1995a) and may be partly based on vago-vagal reflexes to coordinate digestion, including changes in gastric motility and secretion that precede feeding (Rogers et al., 1995). The positive anticipation of the event, combined with the satisfaction of the motivation to feed (Zebunke et al., 2011), may contribute to this autonomic reaction in reactive pigs in period 2 during feeding. Conversely, this vagal dominance was not found in proactive pigs during the consumption of food. The RMSSD values of proactive pigs during food intake even fell below the values during idling (TI 1) before the person was present in both periods. The sympathetic activation with accompanying vagal decrease indicated that proactive pigs were in an aroused state with a rather negative valence during feeding. One possible explanation for this effect may be jealousy over food of the adjacent pen-mates during feeding. Several studies with restrictive feeding during single housing show similar autonomic reactions, namely, a rise in HR as reaction to feeding and an elevated HR during food consumption (Marchant et al., 1997; Geverink et al., 2003). In these studies, HR was elevated during the consumption of food and did not decrease, as found in reactive pigs in the present study. In the study of Marchant et al. (1997), pigs were housed individually, so that competitive interactions were not possible, but still HR remained elevated during feeding. The authors see a major causal factor in the psychologically perceived threat, especially for subordinate pigs housed next to more dominant animals. Additionally, proactive coping strategies were found to be related to higher aggressiveness in several species (Hessing et al., 1994b; Sgoifo et al., 1996). The higher sympathetic activation in proactive pigs during feeding as found in the 5 min analysis was not reflected in the detailed 10 s analysis. This indicated that the sympathetic activation did not occur in the first 30 s of feeding but over the consumption period of 5 min. We consider that the proximity to the trough of the adjacent pen-mate paired with full sight of the feeding conspecific (transparent walls) may explain the higher sympathetic arousal during feeding in proactive pigs.

### Affective Autonomic and Behavioral Reactions during Handling

A different picture regarding autonomic reaction was found during repeated handling by a familiar person. When the person was standing in front of the single pen, both coping styles demonstrated a strong vagal activation with the absence of sympathetic arousal. This reaction was already described in other studies indicating an orienting reflex, which was accompanied by bradycardia of vagal origin serving as information processing of environmental stimuli (Porges, 1995b). A parallel sympathetic activation, which would indicate an anticipation reaction, failed to appear. We assumed that neither coping style clearly anticipated the handling situation. One explanation of this effect may involve the vagueness of the stimulus. As it mainly consists of visual components, some of the pigs might have not detected or identified the person in front of the respective pen in the time window of 10 s. In contrast, the anticipation stimulus during feeding was more pronounced, as it consisted of auditory cues regarding the preparation of food pellets in the buckets.

As the person entered the pen for handling in period 1 (TI 3), HR increased markedly in reactive pigs due to the combined effect of sympathetic activation and vagal withdrawal. This may indicate an emotional state which is characterized by a negative valence and a high degree of arousal. This finding was also supported by a longer latency time to approach the familiar person in reactive pigs. This higher susceptibility to changing environmental conditions in reactive animals is supposed to result in higher behavioral flexibility (Coppens et al., 2010; Koolhaas et al., 2010).

In period 2, the rise in HR and sympathetic activation disappeared in reactive pigs. Instead, reactive pigs showed an elevated vagal tone in TI 3 compared to TI 1 before the person was present. This state is thought to occur in situations with positive emotional valence, such as pleasure or excitement (Boissy et al., 2007). Similar effects were already found in rhesus monkeys demonstrating a rise in vagal tone during grooming carried out by a familiar human (Grandi and Ishida, 2015). The autonomic reaction was also accompanied by a behavioral change in period 2, as the repeated handling procedure decreased the latency time to approach the familiar human in reactive pigs. Several (farm) animal studies demonstrate the importance of human-animal relationships (Waiblinger et al., 2006). It has been demonstrated in pigs, that early handling increased play and exploration behavior, both of which are assumed to be associated with positive emotional states (Zupan et al., 2016). In proactive pigs in period 1, the rise in HR during handling (TI 3–5) was triggered solely by vagal withdrawal as seen in the lack of sympathetic activation. Paired with short approach latencies, we assume that proactive pigs reveal less arousal in response to handling at least in this first experimental period, compared to reactive pigs.

Interestingly, proactive pigs developed strong sympathetic arousal during handling in period 2, which was reflected in a rise of HR compared to period 1 and compared to reactive pigs. Other than during feeding, this sympathetic arousal developed only in period 2 and was not present in period 1. One possible explanation for this effect may include the fact that handling resulted in a style of environmental enrichment, as it can elicit positive emotions by providing stimulations and opportunities for new behaviors. Animals quickly lose interest in simple objects in their environment if they are not relevant (Newberry, 1995). This is not the case for handling, as pigs were highly motivated to explore the familiar person, as indicated by the short approach latencies. Although tactile stimulation by a handler may not necessarily be experienced as positive by the individual, recent physiological evidence using HR and cortisol measurements in pigs (Tallet et al., 2014) suggests that it can be experienced as such. To summarize, reactive pigs seem to develop an increasing state of relaxation with positive emotional valence during a repeated handling situation, whereas proactive pigs were characterized by tachycardia and a strong arousal of the sympathetic nervous system.

## Conclusion

Our study revealed significant context-related differences in the general autonomic reaction of pigs characterized as belonging to different coping styles. More specifically, the two coping styles differ in their affective appraisal in relation to a repeated feeding and handling situation with a familiar person. The present approach contributes insight into the underlying neurophysiological processes of affective appraisal related to distinct coping strategies. The comprehension of these complex relationships is of crucial importance in understanding affect and emotion in the context of human and animal health and welfare.

## Author Contributions

BP, JL and AK contributed to the conception and design of the study; interpreted the data. AK performed the experiments and collected and analyzed the data; wrote the manuscript.

## Conflict of Interest Statement

The authors declare that the research was conducted in the absence of any commercial or financial relationships that could be construed as a potential conflict of interest.

## Abbreviations

ANS: autonomic nervous system
BP: blood pressure
BPV: blood pressure variability
ECG: electrocardiogram
HR: heart rate
HRV: heart rate variability
RMSSD: root mean square of successive differences
SBP: systolic blood pressure
SD_SBP_: standard deviation of systolic blood pressure
TI: time interval.
